# Supplementary figures and images for: Modified W-type configuration for a single-phase reduced parts count 81-level inverter
Source: PLoS One. 2022 Jun 22;17(6):e0269714. doi: 10.1371/journal.pone.0269714 (PMC9216590; doi:10.1371/journal.pone.0269714)

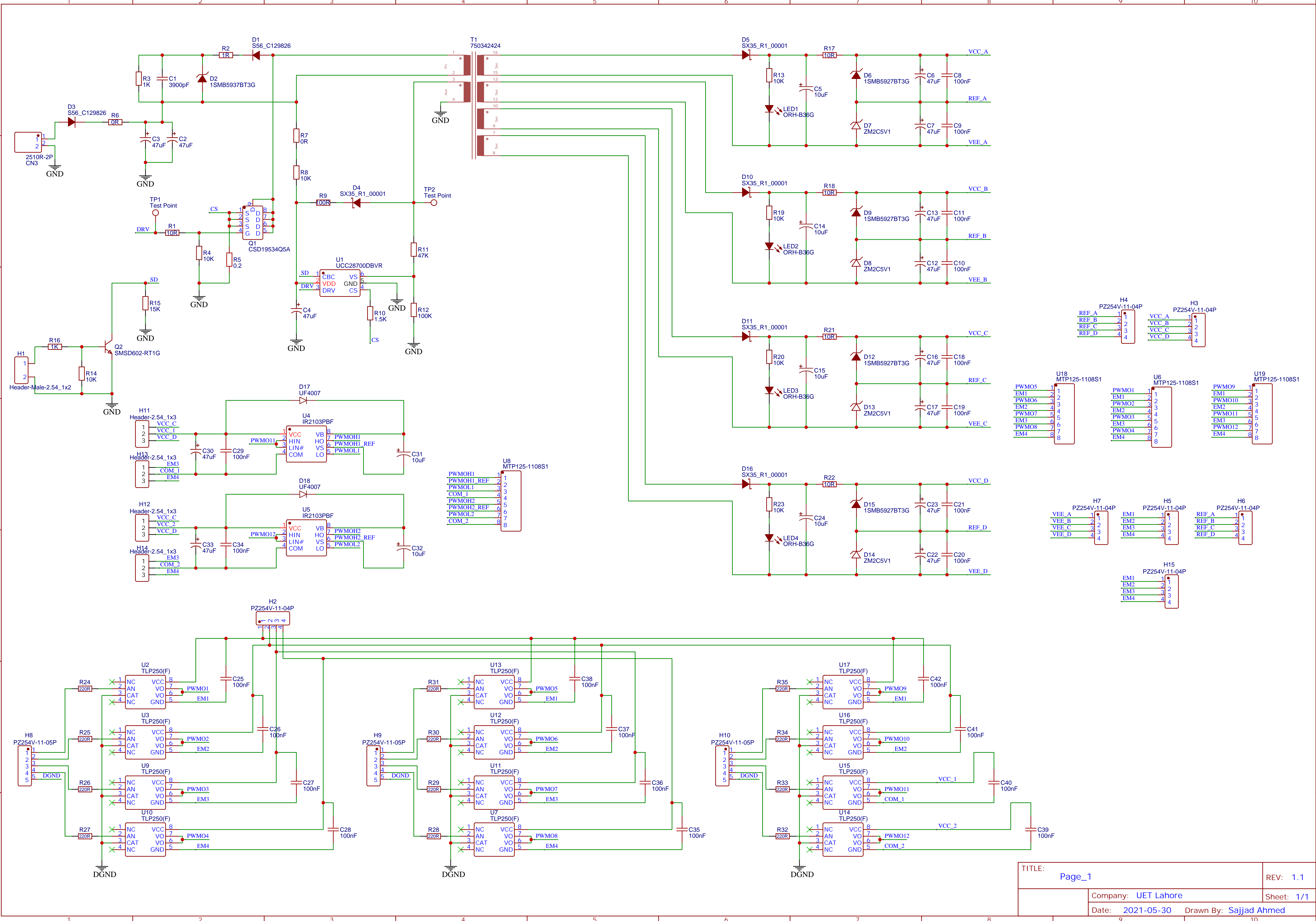

Supplement: S2 Fig — (PDF) [file pone.0269714.s002.pdf]
