# Supplementary material for: Modified W-type configuration for a single-phase reduced parts count 81-level inverter
Source: PLoS One. 2022 Jun 22;17(6):e0269714. doi: 10.1371/journal.pone.0269714 (PMC9216590; doi:10.1371/journal.pone.0269714)
Supplement: Attachment — Submitted filename: Response to Reviewers.docx [file pone.0269714.s003.docx]

**Journal Requirements:**

**Comment#1:** Please ensure that your manuscript meets PLOS ONE's style requirements, including those for file naming. The PLOS ONE style templates can be found at

https://journals.plos.org/plosone/s/file?id=wjVg/PLOSOne_formatting_sample_main_body.pdf and

https://journals.plos.org/plosone/s/file?id=ba62/PLOSOne_formatting_sample_title_authors_affiliations.pdf

Comments: We are thankful to the editorial office for allowing us to resubmit the manuscript.

**Action taken**: We believe that the updated paper is as per PLOS ONE style templates.

**Comment#2:** In your Data Availability statement, you have not specified where the minimal data set underlying the results described in your manuscript can be found. PLOS defines a study's minimal data set as the underlying data used to reach the conclusions drawn in the manuscript and any additional data required to replicate the reported study findings in their entirety. All PLOS journals require that the minimal data set be made fully available. For more information about our data policy, please see <http://journals.plos.org/plosone/s/data-availability>.

Upon re-submitting your revised manuscript, please upload your study’s minimal underlying data set as either Supporting Information files or to a stable, public repository and include the relevant URLs, DOIs, or accession numbers within your revised cover letter. For a list of acceptable repositories, please see http://journals.plos.org/plosone/s/data-availability#loc-recommended-repositories. Any potentially identifying patient information must be fully anonymized.

Comments: We highly thank the editorial team for their interest in the minimal data set underlying the results described in the manuscript.

**Action taken**: All the data for the manuscript is either included within the manuscript or uploaded as supporting information.

**Comment#3:** Please ensure that you refer to Figures 3 to 12, 16 and 20 in your text as, if accepted, production will need this reference to link the reader to the figure.

Comments: We thank the editor for the information about the figures.

**Action taken**: Figures 3 to 12 are referred at line 116. Figure 16 (in the previous version) is now named Figure 17 and is referenced on line 125. Figure 20 (in the previous version) is now named Figure 22 and is referenced on line 148.

**Rebuttal Letter**

We highly thank all the reviewers for their constructive comments to update the manuscript. After incorporating the suggested updates, the manuscript is looking much better than it was before. The response to each reviewer’s comments is given below in order.

**Reviewer#1:** The general idea of the paper seems to be good. However, the paper organization is not acceptable, and there are several major technical challenges that should be effectively addressed.

**Concern#1:** Need more experimental results by varying load, and modulation index.

**Response**: We have added two new experimental waveforms (one for modulation index 0.88 @ 1.0 PF and the other for modulation index 0.85 @ 0.9 PF lagging in the updated manuscript as Fig 24 and Fig 25.

**Concern#2:** Include the efficiency of the proposed inverter.

**Response**: Thank you for suggesting to include efficiency which is an important performance parameter to discuss. The efficiency of the proposed inverter is calculated based on experimental results for 8 different loading conditions (4 different loads @ 1.0 PF and 4 different loads @ 0.8 PF lagging) and included in the ‘Loss and efficiency analysis’ section. A plot of efficiency versus output power for active and reactive load is also included in Fig 26.

**Concern#3:** The abstract has been briefly written and should be enriched by adding the main ideas and contributions.

**Response**: We have updated the abstract of the paper and all the updates are highlighted in yellow color.

**Concern#4:** There are some grammatical errors and typos that should be corrected before publication.

**Response**: We have thoroughly proofread the manuscript and removed all the typos and grammatical errors in the updated paper. We believe that the updated manuscript is free from such grammatical errors and typos.

**Concern#5:** It is recommended to provide a nomenclature at the beginning of the paper to define all variables clearly.

**Response**: As per your suggestion, the nomenclature to define all the variables clearly, is added at the beginning of the paper (before the ‘Introduction’ section) and is highlighted in yellow color.

**Concern#6:** The introduction has been vaguely written. My suggestion is to divide the introduction into three subsections: 1) motivation and incitement, 2) literature review and 3) contribution and paper organization.

**Response**: We believe that the introduction section in the previous version was as per your suggestions. However, we have updated it by adding some more details and highlighting them (line 70-74 and line 79-82).

**Concern#7:** The main contribution of the paper should be highlighted and emphasized. It would be great if the drawbacks and gaps of literature are clear and, particularly, how the proposed approach aims at filling these gaps.

**Response**: We have updated the last few paragraphs of the introduction section. The main contributions are highlighted. We have also clearly mentioned the literature gaps and how the proposed approach fills those gaps. Please refer to lines 70-74 and lines 79-82 in the updated paper.

**Concern#8:** Simulation Results should be enriched. More detailed scenarios should be added.

**Response**: We highly thank the reviewer for his suggestion about adding more simulation results. We have added two new figures (Fig 14 and Fig 19) to the manuscript. Fig 14 shows the zoomed-in voltage levels for the output voltage waveform. Fig 19 shows the effects of dynamic load switching on the output of the inverter.

**Concern#9:** A separate section should be added for discussion of obtained results and main achievements.

**Response**: We have added a separate section for discussion (before the ‘Conclusion’ section) to highlight the main achievements and discussion about the results obtained.

**Concern#10:** The quality of all the figures is poor and should be improved.

**Response**: We have updated all the figures in the updated manuscript to improve the graphics quality.

**Concern#11:** Need to update the following papers and compare with proposed topology.

**Response**: A comparison table (Table 3) shows the comparison of various parameters such as number of levels, number of DC voltage sources, number of switches, number of diodes, number of capacitors, number of gate drivers, components per level factor, THD and TSV of the proposed inverter with the existing multilevel inverter topologies in the literature.

**Reviewer#2:** This paper presents a Modified W-Type Configuration for a Single-Phase Reduced Parts Count 81-Level Inverter; the reviewer has the following comments and questions for the authors to address:

**Concern#1:** Abstract needs to be rewritten to include some significant results.

**Response**: We thank the reviewer for this constructive suggestion as the abstract is the main theme of a manuscript. We have updated the abstract of the paper and all the updates are highlighted in yellow color.

**Concern#2:** More results should be added at different loading conditions/input voltage.

**Response**: We have added more simulation (Fig 14 and Fig 19) and experimental (Fig 24 and Fig 25) results in the manuscript. Fig 19 shows the effect of dynamic load switching on the inverter. Fig 24 and 25 show the output voltage and current waveforms at different loading conditions and modulation index.

**Concern#3:** Add a photo of the experimental setup if available.

**Response**: The photo of the experimental setup has already been included in the manuscript. Please refer to Fig 21.

**Concern#4:** Discussion on the limitation of the proposed MLI topology should be added.

**Response**: We have added a separate ‘Discussion’ section (before the ‘Conclusion’ section) and highlighted all the main achievements and limitations of the proposed inverter configuration.

**Concern#5:** The paper requires further English revision as it has many grammatical mistakes.

**Response**: We have thoroughly revised the paper to remove all the typos and grammatical errors. We believe that the updated paper is free from such mistakes.

**Concern#6:** A detailed comparison should be presented, with what has been presented in the literature ( use a table format with specific parameters such as the number of switches, no. of diodes, passive components….).

**Response**: A comparison table (Table 3) was already available in the submitted paper. However, we have updated it and added more comparisons with existing multilevel inverter topologies.

**Reviewer#3:** Dear Authors, my suggestions to improve the quality of the manuscript are as follows:

**Concern#1:** Authors stated in the abstract, "The proposed topology offers very low total harmonic distortion of the output voltage that is within the benchmarks specified for grid integration". My 1st major concern is to developing a grid-integrated system. If not able to implement experimentally, the simulation results of the same must be incorporated in the revised manuscript.

**Response**: We thank the reviewer for this suggestion. Since the main idea of the paper is to present a novel 81-level inverter configuration with reduced parts count, the idea of a grid-integration system seems to be out of the scope of this paper. The scope of the presented paper is to propose a novel multilevel inverter configuration having reduced hardware parts count to generate an 81-level output voltage that resembles a sinusoidal waveform without the need for an output filter. Therefore, it can meet the grid integration standards about output voltage quality. The configuration discussed in the proposed paper is operating standalone. However, one can use the proposed topology to develop a grid integrated system as the proposed topology has the merit to get integrated with the grid without any filter requirements. We are planning to research on the grid-integration part and we hopefully address this in the next manuscript as currently, it is not the scope of this paper.

**Concern#2:** The second major concern is the availability of DC sources, cost of DC sources, What if the DC sources are replaced by the PV sources for the grid integration. Elaborate the scheme.

**Response**: Since the proposed inverter configuration uses four DC voltage sources and generates an 81-level output voltage by the addition and subtraction of DC voltage sources, any available DC sources whether they are regulated DC voltages of PV systems or obtained from DC-DC converters can be used. The proposed inverter configuration uses four DC sources with a voltage ratio of 1:3:9:27. Therefore, the PV sources along with batteries can be cascaded to obtain higher ratios. The scheme can be elaborated as follows:

Suppose the voltage of 1 PV panel is taken as the lowest DC voltage. The voltages with ratios 3, 9, and 27 can be obtained by cascading other 3, 9, and 27 independent PV sources. Therefore, in total, 40 PV sources will be required along with 40 batteries to run the proposed inverter. The same idea can be implemented in the case of DC-DC converters.

At solar parks where a large number of PV panels and batteries are available, the proposed inverter can be used to generate an 81-level output voltage compatible with the electric power grid.

We have attached the scheme (as a Figure named ‘S1 PV_Scheme.eps’) as a supporting information for obtaining the DC voltages from PV systems.

**Concern#3:** Third concern is about the blocking voltage of the switches. How, the selection of the power switches are made as the DC sources are diverse.

**Response**: Thank you for your comment about the selection of power switches in terms of blocking voltages. The blocking voltage of a switch is the maximum voltage across the switch during the OFF state. It is an important parameter for the selection of power switches used in a given inverter topology. The maximum possible OFF state voltage of all the power switches in the proposed configuration is calculated and represented in terms of multiples of the lowest DC voltage in the circuit.

For example, in the proposed inverter configuration, the switches S_11_, S_12_, S_13,_ and S_14_ can have maximum blocking voltage equal to the value of DC source V_1_ (the lowest DC voltage). The switches S_7_, S_8_, S_9,_ and S_10_ can have a maximum blocking voltage equal to the value of DC source V_2_ (3 times the lowest DC voltage). The switches S_3_, S_4_, S_5,_ and S_6_ can have a maximum blocking voltage equal to the value of DC source V_3_ (9 times the lowest DC voltage). The switches S_1_ and S_2_ can have a maximum blocking voltage equal to the value of DC source V_4_ (27 times the lowest DC voltage). The switches H_1_, H_2_, H_3,_ and H_4_ can have a maximum blocking voltage equal to the sum of all the four DC sources (40 times the lowest DC voltage).

Therefore, any values of DC voltage sources (with ratio 1:3:9:27) can be applied to the proposed inverter after its switches are selected based on the values of the selected input DC voltage sources.

**Concern#4:** The comparison made is not sufficient. Please compare with the papers as similar to following:

Experimental Verification of a New Scheme of MLI Based on Modified T-Type Inverter and Switched-Diode Cell with Lower Number of Circuit Devices

A New and Generalized MLI with Overall Lesser Power Electronic Devices

A new and modular back-to-back connected t-type inverter for minimum number of power devices, tsv, and cost factor

Optimum structure based Multi-level Inverter with doubling circuit configuration

Reduction of power electronic devices in a single-phase generalized multilevel inverter

Analysis and implementation of a generalised switched‐capacitor multi‐level inverter having the lower total standing voltage

A new and generalized structure of MLI topology with half-bridge cell with minimum number of power electronic devices

N-Level Cascade Multilevel Converter with optimum number of switches

Reduction in controlled power switches for a single-phase novel multilevel inverter.

**Response**: We have updated the comparison table (Table 3) by including the comparison with a few of the suggested articles by the reviewer. Please refer to Table 3 and Fig 27-29. We believe that the comparison is now sufficient to verify the novelty of the proposed MWMLI configuration.

**Concern#5:** Fourth major concern is about the loss analysis and experimental results. The experimental results of the dynamic condition are missing. No data for the loss analysis (conduction losses and switching losses) and efficiency is provided. Justify.

**Response**: We updated the ‘Loss and efficiency analysis’ section by adding the experimental efficiency calculation results. One new figure for the experimental efficiency plot versus output power at various loading conditions is added as Fig 26.

**Concern#6:** Include the detailed circuit diagram, operation for the driver circuit and the delay circuit.

**Response**: We have submitted a supporting file (as a Figure named ‘S2 Gate_Driver.pdf’) that includes the detailed circuit diagram of the gate driver circuit.
