# Supplementary material for: Modified W-type configuration for a single-phase reduced parts count 81-level inverter
Source: PLoS One. 2022 Jun 22;17(6):e0269714. doi: 10.1371/journal.pone.0269714 (PMC9216590; doi:10.1371/journal.pone.0269714)
Supplement: Attachment — Submitted filename: Response to Reviewers.docx [file pone.0269714.s004.docx]

**Journal Requirements:**

**Comment#1:** Please review your reference list to ensure that it is complete and correct. If you have cited papers that have been retracted, please include the rationale for doing so in the manuscript text, or remove these references and replace them with relevant current references. Any changes to the reference list should be mentioned in the rebuttal letter that accompanies your revised manuscript. If you need to cite a retracted article, indicate the article’s retracted status in the References list and also include a citation and full reference for the retraction notice.

Comments: We are thankful to the editorial office for allowing us to resubmit the manuscript after minor revisions.

**Action taken**: We have reviewed the reference list (one by one) of the manuscript to ensure that it is complete and correct. We are confident that all the cited papers are available in the literature.

**Additonal Editor Comments:** One reviewer has asked the authors for thoroughly proofreading of the paper before final acceptance. Hence, authors are advised to do the same with sincerity and effectively.

**Action taken**: We have thoroughly proofread the manuscript and removed all the typos and grammatical errors in the revised version of the paper. We believe that the revised manuscript is free from grammatical errors and typos.

**Rebuttal Letter**

We highly thank all the reviewers for their constructive comments to update the manuscript. After incorporating the suggested updates, we are confident that the revised manuscript will meet the publication criteria. The response to each reviewer’s comments is given below in order.

**Reviewer#1:** Authors may include more details about the applications because this single phase system will not suitable for high power applications. All my comments are addressed no more questions.

**Response**: We thank the reviewer that he/she is satisfied with our previous revision. We have added a few lines (highlighted in yellow color) in the ‘Conclusion’ section to highlight more details about the applications of the proposed single-phase inverter system.

**Reviewer#2:** Most of the comments have been addressed, just need to do proofreading before acceptance.

**Response**: We have thoroughly revised the paper to remove all the typos and grammatical errors and we believe that the updated paper is free from such mistakes.

**Reviewer#3:** Dear Authors, The paper now can be accepted in its current form. The comments have been addressed.

**Response**: We are highly obliged for your recommendations about our manuscript.
